# Zebrafish Models for Ectopic Mineralization Disorders: Practical Issues from Morpholino Design to Post-Injection Observations

**DOI:** 10.3389/fgene.2013.00074

**Published:** 2013-05-08

**Authors:** Mohammad Jakir Hosen, Olivier M. Vanakker, Andy Willaert, Ann Huysseune, Paul Coucke, Anne De Paepe

**Affiliations:** ^1^Center for Medical Genetics, Ghent University HospitalGhent, Belgium; ^2^Department of Genetic Engineering and Biotechnology, Shahjalal University of Science and TechnologySylhet, Bangladesh; ^3^Department of Biology, Ghent UniversityGhent, Belgium

**Keywords:** zebrafish, embryos, morpholino, mineralization, osteogenic pathways

## Abstract

Zebrafish (ZF, *Danio rerio*) has emerged as an important and popular model species to study different human diseases. Key regulators of skeletal development and calcium metabolism are highly conserved between mammals and ZF. The corresponding orthologs share significant sequence similarities and an overlap in expression patterns when compared to mammals, making ZF a potential model for the study of mineralization-related disorders and soft tissue mineralization. To characterize the function of early mineralization-related genes in ZF, these genes can be knocked down by injecting morpholinos into early stage embryos. Validation of the morpholino needs to be performed and the concern of aspecific effects can be addressed by applying one or more independent techniques to knock down the gene of interest. Post-injection assessment of early mineralization defects can be done using general light microscopy, calcein staining, Alizarin red staining, Alizarin red-Alcian blue double staining, and by the use of transgenic lines. Examination of general molecular defects can be done by performing protein and gene expression analysis, and more specific processes can be explored by investigating ectopic mineralization-related mechanisms such as apoptosis and mitochondrial dysfunction. In this paper, we will discuss all details about the aforementioned techniques; shared knowledge will be very useful for the future investigation of ZF models for ectopic mineralization disorders and to understand the underlying pathways involved in soft tissue calcification.

## Introduction

Mineralization is an essential step in the process of skeletal tissue formation, which needs to be rigorously controlled and restricted to specific regions. Though incompletely understood, multiple factors – both agonists and inhibitors – have been shown to work synergistically in achieving physiological mineralization of bone and dental tissue (Hosen et al., 2012). In many cases, ectopic mineralization results from a disturbance of the complex interplay between mineralization propagators and antagonists set out to regulate this process (Shanahan et al., 2011). Although most soft tissues can undergo calcification, certain ones – including skin, kidneys, cartilage and tendons, eyes, and vasculature – are considerably more prone. Pathologic mineralization in any of these tissues may result in disease with significant morbidity and mortality (Viegas et al., 2009). The mechanisms contributing to physiological calcification not only play a pivotal role in the pathophysiology and clinical manifestations of these diseases, but also in the prognosis and possible therapeutic options for rare as well as more common calcification disorders.

Traditionally, mice have been the preferred models of human diseases, often through the development of Knockout (KO) animals by targeted ablation of the corresponding genes. While the KO mice often show remarkable similarity to the human phenotype both at the genetic, gross morphologic, histopathologic, and ultrastructural level, this model system knows considerable limitations (Lieschke and Currie, 2007). It may take several years to develop a KO mouse and in some cases, development of a KO mouse of the corresponding human disease is not feasible due to the absence of the corresponding gene in the mouse genome such as the human SMAD9 gene (Li et al., 2007). These considerations, together with cost containment, the large space needed to keep the animals and low number of progeny, has prompted the search for alternative model systems to study mineralization-related disorders.

The zebrafish (ZF, *Danio rerio*), a freshwater vertebrate belonging to the teleosts, has become an important model for the study of basic pathogenetic mechanisms in different human diseases. The popularity of the ZF as a model organism is due to some important properties including their easy maintenance in the laboratory at low cost, the production of large numbers of synchronous developing embryos per mating, the short reproductive cycle with external fertilization and rapid rate of development, the optical transparency of the embryos, and the possibility to conduct high resolution *in vivo* imaging, availability of a wide range of molecular techniques such as large-scale genome mutagenesis and over-expression/knock-down approaches, and freely available web based resources, i.e., ZFIN, Zebrafish International Resource Center (ZIRC), Trans-NIH ZF initiative, ZF-Health, etc.

The ZF genomic sequence, which was completely mapped in 2009 (Bhartiya et al., 2010), demonstrates about 70% homology with the human genome, suggesting the evolutionary conservation of a large number of genes and genetic pathways. Due to an ancient genome duplication event that occurred after divergence of actinopterygian and sarcopterygian ancestors, a substantial number of ZF genes are present in two or more copies (Woods et al., 2000; Lu et al., 2012). Although the genome duplication can complicate gene function analysis, in some cases, this resulted in the partitioning of ancestral gene functions between duplicated descendants. This role-separation by paralogs can provide a unique opportunity to study the sub-functions of an individual human gene (Postlethwait et al., 2004).

To explore physiological and aberrant (soft tissue) mineralization in ZF, till now the bone, skin, scales, and vasculature have been intensively studied. ZF have been proposed as a model to study osteogenesis, bone metabolism, and remodeling based on study of their scales, although these structures are strictly speaking tooth- and not bone-related (Sire and Akimenko, 2004; Pasqualetti et al., 2012). Bone mineralization is an essential and well-orchestrated process in vertebrates in which crystals of calcium phosphate are orderly arranged into hydroxyapatite crystals in very close association with collagen fibers to build the bone mineralized matrix (Kawasaki et al., 2009). The ZF skeleton shows high similarity with human bones in terms of cells, matrix proteins, and molecular signaling pathways involved. The latter include Notch, Wnt, and TGF beta/bone morphogenetic proteins (BMP) signaling pathways (Rusanescu et al., 2008).

The skin of ZF consists of two layers: the epidermis and the dermis. At 1 day post fertilization (dpf), two different layers representing the epidermis and the dermis can be recognized. At 6 dpf, a two cell layer epidermis can be observed, clearly separated from the underlying connective tissue stroma (Li et al., 2011). Despite several structural differences (lack of keratinization, presence of several unicellular glands, etc.), the epidermis of the ZF shows high homology with the human epidermis, and several similar molecular genetic factors and mechanisms – including the retinol-binding protein 4 (RBP4) and apolipoprotein Eb (APOEB) – were reported in both species in the process of (epi)dermal development and homeostasis (Tingaud-Sequeira et al., 2006). However, the underlying molecular mechanisms of scale calcification which occur during ZF development are still incompletely understood. The scales of ZF, represent a significant reservoir of calcium and are subjected to a specific mineralization pattern. In elasmoid scales, deposition of mineralization-related proteins occurs from the epidermis into the dermis (Sire et al., 1997a,b; Hong et al., 2011). Besides detailed morphological studies, characterization of the molecular events involved in scale development has been initiated, revealing the role of Sonic Hedgehog (Shh), BMP2, 4, osteocalcin, or osteopontin (Sire and Akimenko, 2004; Hong et al., 2011).

Because of the transparency of the embryonic stages, *in vivo* observation of the heart rhythm as well as the vasculature and circulation in ZF is possible and does not require physical intervention. The heart of ZF embryo starts beating within 26 hour post fertilization (hpf; Baker et al., 1997) and undergoes looping by 2 dpf (Stainier et al., 1996). A fully functioning vascular tree is present by 3 dpf (Sehnert and Stainier, 2002). At 4 dpf, cardiomyocyte proliferation thickens the ventricular wall (Antkiewicz et al., 2005) and by 5 dpf the heart has developed valves (Forouhar et al., 2004). To characterize the large number of cardiovascular mutants in ZF, a comprehensive array of cellular, molecular, physiological, and genetic techniques has been developed (Warren and Fishman, 1998; Xu et al., 2002). The vital nature of the heart poses a challenge for studying its function at a molecular level. Although targeted inactivation of mouse genes can provide a wealth of information, inactivation of heart-specific genes is frequently hampered by the early embryonic lethality it creates. By contrast, the ability of ZF embryos to survive on diffused oxygen for several days without a functioning cardiovascular system is an important distinguishing feature. Using ZF, forward genetic screens have been conducted, identifying more than 100 genes required for heart formation and function (Chen et al., 1996; Stainier et al., 1996). Studies in ZF, mice, and humans indicate that Notch works in conjunction with other angiogenic pathways to pattern and stabilize the vasculature (Shawber and Kitajewski, 2004). Recent progress in cardiovascular research has suggested that arterial and valve calcification is the result of an active process of osteogenic differentiation, induced by pro-atherogenic inflammatory response. At the molecular level, the calcification process is regulated by a network of signaling pathways, including Notch, Wnt, and TGFβ/BMP pathways, which control the master regulator of osteogenesis CBFA1/Runx2 (Rusanescu et al., 2008).

One of the most substantial features of ZF in their use as a model system to study ectopic mineralization is that the expression of specific genes can be easily knocked down by injection of 1–4 cell embryos with morpholino (MO)-based anti-sense oligonucleotides (Eisen and Smith, 2008). After MO injection and analysis of its efficacy, a larger number of embryos and larvae can be comprehensively screened for phenotypic manifestations using light microscopy. Once the phenotype has been characterized, the embryos are readily amenable to further investigation (Table 1) at the molecular and cellular level, including calcification-specific stains [i.e., calcein staining, Alizarin Red (AR-S) Staining, Alcian blue-Alizarin red double staining], the use of mineralization specific transgenic lines, and analysis of ectopic calcification-related marker gene expression by quantitative real-time polymerase chain reaction (qPCR) or western blot analysis, respectively. Also specific pathophysiological mechanisms involved in aberrant mineralization, such as apoptosis and mitochondrial dysfunction can be assessed [e.g., using transferase dUTP nick end labeling (TUNEL) and MitoTracker staining]. Hitherto, the number of reports applying MO injection to study (ectopic) mineralization processes and diseases is relatively scarce. Li et al. (2010) were able to KO about 84% of the abcc6a gene [a homolog gene of human ABCC6, causing pseudoxanthoma elasticum (PXE)] and suggested that abcc6a may also have a developmental role. Hughes et al. (2004) knocked down the otop1 (otopetrin) gene required for the formation of otoliths – large extracellular biomineral particles involved in transducing sound into neuronal signals – in the ZF ear, and with more than 96% of otop1 morphants failing to develop otoliths, demonstrated that otopetrin 1 has a conserved role in the timing and shaping of otolith formation. In this paper, we highlight several experimental procedures which can be used to assess ectopic calcification and its related processes in ZF, illustrated with current knowledge on ZF mineralization.

**Table 1:** **Overview of different methods that can be applied in zebrafish models for ectopic mineralization**.

Methods	Application	Stages of application in MO approach
MO injection	To evaluate the gene function by injecting synthetic anti-sense nucleotide oligomers	1–4 Cell stage embryos
Light microscopic observation	Phenotypic screening after injection (see Table 2).	Post-injection to morphant death
RNA rescue experiment	Validation of gene specificity by co-injection of MO and mRNA (encoding protein from the targeted locus of other species	1–4 Cell stages of embryos
Western blotting	Validation of the efficiency of TB MOs	After phenotypic confirmation, 1–4 dpf, until when effect of MO can be observed
PCR	Expression profiling of targeted gene	From 0- different time points
	Validation of the efficiency of SJ MOs	After phenotypic confirmation, 1–4 dpf, until when effect of MO can be observed
Quantitative real-time PCR	Expression profiling of targeted gene	From 0- different time points
	Validation of the efficiency of SJ MOs	After phenotypic confirmation, 1–4 dpf, until when effect of MO can be observed
Calcein staining	Fluorescent chromophores specifically bind to the calcified skeleton of live ZF embryos	5 dpf to morphant death
Alizarin red S	To identify calcium in tissue sections or whole mount embryos	4 dpf to morphant death
Alcian blue-Alizarin red double staining	Alcian blue stains cartilage blue and is used as a counterstaining to AR-S to distinguish cartilage and bone	4 dpf to morphant death
	Alizarin red stains as red in calcified matrix (calcified cartilage, bone)	
IHC	To detect presence and localization of (mineralization-related) protein in tissue sections or whole mount embryos	0 hpf to morphant death
μCT imaging	Useful for skeletal analysis, used to understand developmental processes of three-dimensional embryos, embryo phenotyping, and quantitative modeling of development	5 dpf
ISH	To assess gene expression profiling in wild-type embryo and differential gene expression in morphant	0–4 dpf of embryos, as until 4 dpf effect of MO can be observed
MS	To analyze differential protein expression by measuring the mass-to-charge ratio	0–4 dpf, until MO effect can be observed
2D gel electrophoresis	To assess differential protein expression, where proteins are separated in the gel according to their isoelectric point	0–4 dpf, until MO effect can be observed
Microarray	Used to identify genome-wide expression of genes. In morphant differential expression of different gene can be identified	0–4 dpf, until MO effect can be observed
Transcriptome analysis	More sensitive compared to microarray, used to identify differential expression of transcripts. By this method closely homologous genes can be distinguished, alternatively spliced transcripts and non-coding RNAs can be characterized, and rare transcripts which are undetectable in microarray analysis can be detected	0–4 dpf, until MO effect can be observed
TUNEL staining	To assess *in situ* cell death in the whole mount embryo. TUNEL labels degraded DNA products enzymatically or by a fluorescent probe and stains apoptotic bodies	30 hpf–4 dpf, until MO effect can be observed
CMH2DCF staining	Used to determine oxidative stress or level of ROS in live embryos	0–4 dpf, until MO effect can be observed
MitoTracker Red CM-H2XRos	Used to determine mitochondrial membrane potentiality in live embryo	0–4 dpf, until MO effect can be observed
Chemical screening	Used to identify small chemicals which can rescue the morphant phenotype and can be predicted as a potential drug	0–4 dpf, until MO effect can be observed

*MO, morpholino; PCR, polymer chain reaction; IHC, immunohistochemistry; ISH, *in situ* hybridization; MS, mass spectrophotometry; 2D, 2 dimensional*.

## Gene Knock-Down Approaches

Zebrafish has already been proven a good model for forward (phenotype driven) genetic approaches, where mutagens are employed to produce random changes in the DNA followed by phenotypic evaluation. The unbiased nature has made this approach very powerful (Lieschke and Currie, 2007). Less attention has been given to reverse (candidate gene-driven) genetic approaches, though these have become robust tools when forward genetics is less feasible or to investigate redundant gene function. The ability to make precise targeted changes to a genome has long been the holy grail to the reverse genetic approach. ZF genomic sequencing made it possible to analyze its genes function in a systematic way by inactivating protein-coding genes by targeted or random mutation (Varshney et al., 2013a). The main obstacles of these reverse approaches include the need for available knowledge of candidate genes and the focus on a single gene or a small group of genes. Recently Varshney et al. (2013b) performed proviral insertions coupled with high-throughput sequencing and succeeded to widely mutagenize genes in the ZF genome. To facilitate such studies, the Zebrafish Insertion Collection (ZInC)^1^ have generated a genome-wide KO resource that targets every ZF protein-coding gene (Varshney et al., 2013a). All mutants from ZInC are freely available through the ZIRC. The most frequently used reverse genetic technique is morpholino-induced knock-down. The specificity of the observed phenotypic effects have always been a concern using this technology and can be addressed by running a second or more experiments, applying other reverse genetic approaches as detailed below.

### Anti-sense approaches

#### Morpholino-induced knock-down

**Figure 1:**
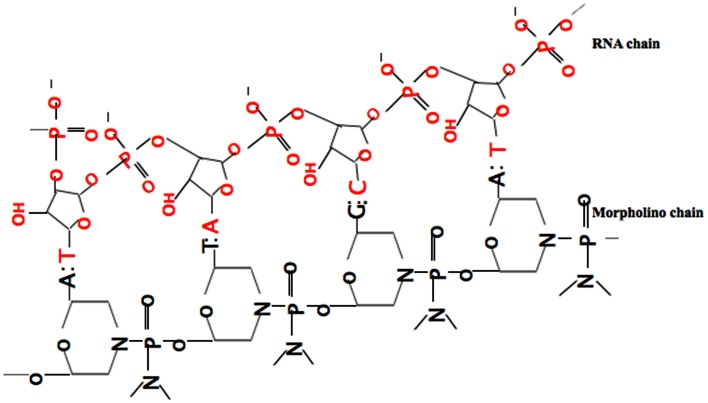
**Binding of the anti-sense morpholino chain to the RNA chain**. MOs have standard nucleic acid bases bound to morpholino ring, which are linked through phosphorodiamidate groups, while RNA has ribose rings which are linked with phosphates.

Among many reverse genetic approaches, the most commonly used method is a gene knock-down approach by injecting so-called morpholinos (MOs). The ease of use and exciting results within few hours made this approach increasingly popular for gene function analysis. MOs are synthetic anti-sense nucleotide oligomers used to block proper gene expression by binding to complementary sequences of RNA. Instead of degrading the target molecules, the knock-down effect is achieved by preventing cells from making the targeted proteins (Summerton, 1999; Nasevicius and Ekker, 2000). Among all gene knock-down reagents, MOs are the only molecules which have all properties of stability, nuclease-resistance, efficacy, comparatively long-term activity, water solubility, low toxicity, and specificity. MOs consist of standard nucleic acid bases (Figure 1) but, contrary to nucleic acids, those bases are bound to morpholine rings and linked through phosphorodiamidate groups (Summerton and Weller, 1997). To date, translational blocking (TB) and/or Splice Junction (SJ) MOs are commonly used. TB MOs can be used to target sequences around or slightly upstream from the translation initiation codon, which interfere with the formation of the ribosomal initiation complex from the 5′ cap to the start codon (AUG) to prevent translation of the coding region of the targeted transcript (Draper et al., 2001). MOs targeted to SJs will modify pre-mRNA splicing or can block the binding sites of splice-regulatory proteins (Bruno et al., 2004).

To obtain ZF embryos for MO injection, adult males and females can be put in breeding tanks and kept separated to control the laying time, by using a separator in the tank. After the divider in the breeding tank has been removed, the fish will usually begin to lay eggs within approximately 30 min, though this period may be extended. To get the most prominent effect of the MO, injection should be done in the 1-cell stage (which allows best distribution of MO in each cell and avoid mosaic phenotypic features). However, MO injection can be done between the 1- and 8-cell stage and still give ubiquitous delivery (Nasevicius and Ekker, 2000). Rapid delivery of MO into embryos is done by MO injections using a glass micro-needle and a micromanipulator with air pressure. An optimal concentration (<6 ng/nl) of MO is preferred to obtain the most specific phenotype (Rikin et al., 2010).To overcome possible redundancy between different paralogs, combined MO injection targeting the different paralogs can be necessary to obtain the expected phenotype (Bill et al., 2009; Bedell et al., 2011). For polygenic disorders, simultaneous injection of different MOs may allow the inactivation of more than one gene at the same time, which represents a paramount advantage, compared to any mammalian assay available and can be exploited for the identification of novel gene(s) (Nicoli and Presta, 2007).

Different procedures are available for the maintenance of injected and wild-type embryos (Lawrence and Mason, 2012). In our lab, embryos are maintained in an E3 medium (containing methylene blue) in Petri dishes and kept in an incubator at 28°C (Kimmel et al., 1995) until 9 dpf for normal growth. Every day, half of the E3 medium is replaced by fresh E3 medium and from 6 dpf on the embryos are fed twice a day with 50 μM solid dry granules.

To avoid melanin formation in the skin, which can hide some internal structures from microscopic observation [phenylthiourea (PTU), a tyrosinase inhibitor commonly used to block pigmentation] can be added starting after gastrulation (around 10–12 h) and before 24 hpf of embryonic life (Renaud et al., 2011). PTU affects early embryonic development, therefore it is important not to treat the embryos with PTU before the end of gastrulation (Thisse and Thisse, 2008).

### RNA interference

RNA interference (RNAi) is a powerful approach to knock-down gene function in different model systems. However, success of RNAi in ZF is not obvious due to ineffective penetrance or non-specific effects (Zhao et al., 2008). Simple small-hairpin RNA (shRNAs)-mediated knock-down approaches also appear ineffective in ZF (Wang et al., 2010). Micro-RNAs (miRNAs) are endogenous ∼21–23 nt RNAs which can also regulate gene expression. Recently, miR-shRNAs were reported to be (12-fold) more efficient in ZF gene knockdown (De Rienzo et al., 2012). However, the capability of germ-line transmission is very poor. Therefore, the potential of RNAi for stable and conditional gene knock-down in ZF remains uncertain.

### Engineered nucleases

#### Transcription activator-like effector nucleases

Transcription activator-like effector nucleases (TALENs) are a powerful and robust approach for efficient inactivation of a targeted gene. TALENs are artificial restriction enzymes generated by fusing a TAL effector (derived from plant pathogenic bacteria Xanthomonas) DNA-binding domain to a DNA-cleavage domain or *Fok*I nuclease. These restriction enzymes are introduced into cells to edit the genome *in situ*. When fused to the *Fok*I nuclease domain, TAL effectors recognize specific DNA sequences using a straightforward DNA base recognition code. *Fok*I cleaves only when present as a dimer and the binding of two TAL effectors to DNA, which allows *Fok*I to dimerize, will thus create a break in the nucleic acid strand. Because of the specificity of this DNA-binding, TAL effectors can bind virtually any DNA sequence (Cermak et al., 2011). TAL effectors possess several advantages, including ease of design and easier optimization, shows few off-target effects and are thus potentially more reliable compared to other reverse genetic approaches (Clark et al., 2011).

#### Zinc finger nucleases

Another nuclease-based technology for efficient inactivation of a targeted gene in ZF (Sander et al., 2010, 2011) is the use of zinc finger nucleases (ZFNs). These are artificial restriction enzymes generated by fusing engineered zinc finger DNA-binding domain to a DNA-cleavage domain, and function as dimmers to introduce targeted DNA double-strand breaks. ZFN treated ZF can transmit mutant alleles (as if they were heterozygous carriers) and cause minimal collateral damage to the genomes of treated ZF. Moreover, it is possible to also generate targeted knock-ins in ZF using ZFNs. However, injected nucleases can be toxic to the embryo (Doyon et al., 2008; Meng et al., 2008) and together with the gaps in our understanding of sequence-specific DNA recognition by zinc fingers can still restrict the ability to construct ZFNs targeting any desired site in a genome (Lawson and Wolfe, 2011).

### Targeting induced local lesions in genomes

Targeting induced local lesions in genomes (TILLING) is a quick, reliable, and increasingly popular method to identify chemically induced point mutations in ZF (Wienholds et al., 2003). The TILLING method combines mutagenesis (by a chemical mutagen) with a sensitive DNA screening-technique that identifies single base mutations in a target gene. The TILLING method relies on the formation of DNA heteroduplexes between the wild type and mutant PCR fragment, formed when multiple alleles are amplified by PCR and are then heated and cooled. These heteroduplexes are then incubated with celery derived CelI endonuclease, which recognizes and cleaves mismatches in the heteroduplex DNA generated by small nuclear polymorphisms (SNPs) and point mutations (Oleykowski et al., 1998). Labeled digested fragments are separated and visualized on slab gel sequencers. Fragments generated due to the presence of SNPs will be present in all animals. This approach holds great promise for the rapid identification of large numbers of mutant alleles from mutagenized libraries. However, the need for screening large number of F1 fish to find a lesion in a gene of interest (Wienholds et al., 2002), the requirement of specialized equipment, and a significant investment in computational resources and personnel represent the initial bottleneck of this approach. To date, the “Welcome Trust Sanger Institute” has successfully provided 8402 genes (including abcc6) with mutations under the “Zebrafish Mutation Project (ZMP) KOs for disease model” by using the TILLING approach^2^.

### Retroviral and transposon mediated mutagenesis

Insertional mutagens retrovirus or transposon can be utilized to identify modified alleles of a target gene. The main advantage of these systems is readily identifiable tag that simplifies screening for carriers of a particular disrupted allele within the library (Jao et al., 2008). It is also possible to generate conditional alleles in ZF by including recombination sites flanking these gene-breaking elements (Petzold et al., 2009). A limitation of these insertion mutagenesis techniques is the inability to generate full null alleles in most instances.

### Dominant negative approach

Here, a mutant gene product is used to adversely affect the wild-type gene product within the same cell to get a reduced level of gene activation. Two main Dominant negative (DN) approaches are: (1) mutation in a transcription factor that removes the activation domain, which can block the wild-type transcription factor to bind with the DNA-binding site, resulting in a reduction of gene activation, and (2) overexpression of a constitutively active protein (CAP) with mutation in/manipulation of the regulatory domain, leading to diminished opportunity for the regulatory subunit of the wild-type protein to bind with its receptor, and binding of a malfunctioning protein domain to the receptor will decrease the wild-type protein expression (Concordet et al., 1996). For example, in a protein which is functional as a dimer, a mutation leading to removal of the functional domain while retaining the dimerized domain would cause a DN phenotype. Lanham et al. (2011) successfully used aryl hydrocarbon receptor 2 (Ahr2) as a DN approach in ZF to protect developing ZF from dioxin toxicity by removing the C-terminal transactivation domain or replacing it with an inhibitory domain. CAP also been used to explore the function of various components of signal transduction pathways. The main advantage of DN approach over anti-sense RNA strategies is the possibility of producing null mutations. The disadvantages of DN approaches are that the technique is not applicable to all genes, has a relatively low throughput, and that it is usually impossible to fully understand the true endogenous function of the molecule *in vivo* (Niwa and Slack, 2007).

### Pharmacological approaches

Pharmacological approaches (PA) are not true reverse genetic approaches, because they do not depend on specific knowledge of an individual gene of an organism. Once the treatment with a reagent reveals phenotypic consequences, this can be utilized to understand interacting molecular pathways. Many pharmacological reagents are used in the ZF model system including, e.g., cyclopamine – an inhibitor of Shh signaling pathway – (Cooper et al., 1998) or SU5614 and SU1498-an inhibitor of VEGF/Flk-1 tyrosine kinase signaling (Liang et al., 2001). Biochemical characterization of the pharmacological reagents is highly recommended before applying them in the model system. Exert pleiotropic effects is the bottleneck of using pharmacological reagents which can result an unspecific phenotype (Skromne and Prince, 2008).

### Inducible systems

Most of the aforementioned techniques used in the ZF gene-knock-down process are only suitable to evaluate phenotype and gene function in early embryonic stages, as severe malfunction of embryo leads to early death. So, gene function(s) at late stages are missed, which can be overcome by inducible systems (IS) including heat shock promoters orgal4-UAS system. Application of IS can be done by regulating the activity of the protein or regulating the expression of the corresponding gene. In ZF, downstream gene expression can be induced throughout the body by raising the temperature from 28.5 to 38°C (Shoji and Sato-Maeda, 2008). Heat shock protein (hsp) promoters have been used to regulate exogenous gene expression in a variety of studies. The hsp promoter can be used to search cis-acting transcriptional elements during ZF embryogenesis. Hsp:egfp (enhanced green fluorescence protein) can be injected into fertilized eggs with distal DNA fragments for tissue/cell type specific activation (Islam et al., 2006). One such way for inducing heat shock response in targeted cell is by using a laser microbeam under the microscope. The main advantage of this technique is visualization of a whole cell due to stable and longtime expression of the GFP protein, however laser ablation can be sensitive to the cells.

## Post-Injection Follow-Up

### Evaluation of the phenotype

**Figure 2:**
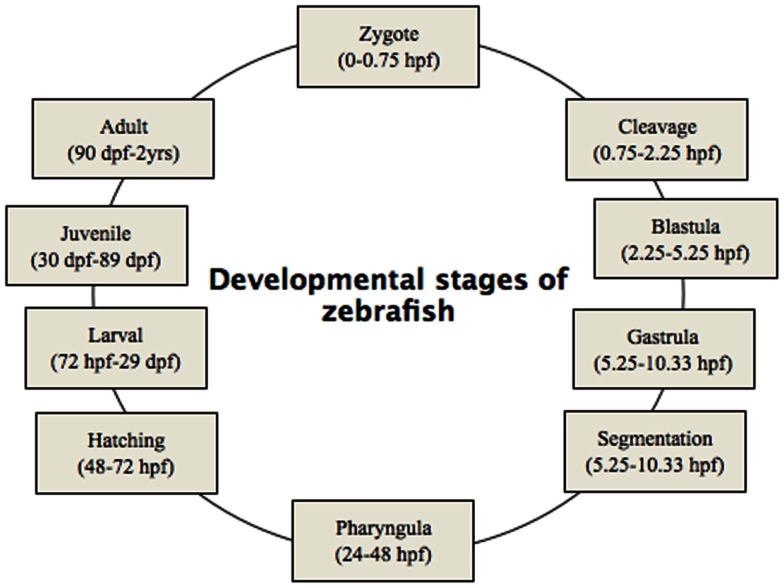
**Developmental stages of zebrafish, from zygote to adult**. Zygote: the newly formed fertilized egg after completion of the first zygotic cell cycle. Cleavage: zygotic cell cycles 2–7 occur rapidly and synchronously. Blastula: rapid and metasynchronous cell cycles (8, 9) occur, which give way to lengthened, asynchronous ones at the midblastula transition, then epiboly begins. Epiboly is the first coordinated cell movement in zebrafish embryos and begins before gastrulation. Gastrula: morphogenetic movements of involution, convergence, and extension from the epiblast, hypoblast, and embryonic axis through the end of epiboly occur. Bud-100% epiboly is the stage where epiboly completely covers the yolk plug. Segmentation: Somites (after completion of epiboly and initial appearance of the tail bud, first the somatic furrow forms and makes a boundary, between what will become the first and second somites), pharyngeal arch primordia, and neuromeres develop, primary organogenesis and earliest movements take place, and the tail appears. Pharyngula: phylotypic stage of embryo, body axis straightens from its early curvature around the yolk sac; circulation, pigmentation, and fins begin development. Hatching: completion of rapid morphogenesis of primary organ systems, cartilage development in head and pectoral fin, hatching occurs asynchronously across individuals. Larval: swim bladder inflates; food-seeking and active avoidance behaviors.

Phenotypic screening is mainly done based on classic criteria (Haffter et al., 1996), which can provide important information on gene function as well as the molecular events that underlie a biological process. Such screening focuses primarily on morphological landmarks by dissecting microscopy. A significant number of phenotypic traits can be screened for, as summarized in Table 2. The phenotypic screening is conducted at different time points corresponding to the embryonic developmental stages (Figure 2). Due to transient character of MO-induced gene knockdown, with efficient knockdown up to 4–5 dpf, phenotypic screening is preferably done during this time frame (Nasevicius and Ekker, 2000; Bill et al., 2009).

**Table 2:** **Phenotypic traits which can be screened in ZF embryos beyond early development**.

System	Phenotypic trait
Body axis	Dorsilisation (48 hpf)
	Ventrilisation (48 hpf)
	Prechordal plate and hatching (24 hpf)
	Tail (24–48 hpf)
Mesoderm	Notochord formation, differentiation, and degeneration (24 hpf)
	Somite formation and patterning (24 hpf)
Central nervous system	Forebrain (24 hpf)
	Midbrain (24 hpf)
	Hindbrain (24 hpf)
	Neural tube – spinal cord (24 hpf)
Organs	Vasculature (e.g., aortic arches, dorsal aorta, common/posterior cardinal vein, blood island; 24–48 hpf)
	Heart: morphology, beating (48 hpf)
	Liver, kidney, gut (larva stadium)
	Eye (24 hpf)
	Ear (48 hpf)
	Otoliths (48 hpf)
Pigmentation	Cell number and pattern (48 hpf)
	Melanin pigmentation (48 hpf)
Motility	Muscles (48 hpf)
	Pectoral and caudal fin (48 hpf – larva stadium)
	Reduced motility (48 hpf)

*For each trait, the timing when evaluation can start is mentioned. From this timing on, serial evaluations in time are usually performed*.

Morpholino mediated knockdown to study gene function in ZF offers many advantages, including ease of delivery, a high efficacy, and rapid phenotypic screening. However, because of their dilution in rapidly growing embryos, the effect of MOs is temporary, preventing gene function analysis during the entire life cycle of ZF. Moreover, many genes – e.g., the macrophage stimulating protein Msp (recently found to have a role in the regulation of calcium homeostasis of adult ZF) (Huitema et al., 2012) – which are expressed during the adult period and some organs such as the skeleton which have only fully matured after 2–3 weeks cannot be studied sufficiently after MO knockdown. Altogether, this emphasizes the usefulness of permanent mutants in addition to MO-induced models.

### Morphant validation

Every MO-induced phenotype must be validated to confirm that it is due to gene-specific effects. Besides the application of an independent knock-down approach as described above, several MO-specific validation steps need to be addressed. First, to assess the phenotypic variation and effect of the injection procedure, commercially available standard control MO needs to be injected in parallel with the active MO. Secondly, for each targeted gene, the injection of both TB and SJ MO is recommended, to reveal the role of either the entire protein-coding region of the gene, or of certain exons. MOs appear to have non-target-related phenotypes including overall developmental delay and cell death due to activation of p53-mediated apoptosis (Ekker and Larson, 2001), which can be overcome by using stage matched (rather than age matched) control embryos and co-injection of an anti-p53 MO along with the experimental MO. Injection of SJ MO generally causes skipping of the targeted exon or retention of the adjacent intron while TB MO blocks gene transcription. These molecular effects can be validated by conducting western blotting in case of SJ MO’s and RT-PCR for TB MO’s. Further evidence of the specificity of the gene knockdown can be obtained by rescuing the phenotype by mRNA injection.

#### RNA rescue experiments

mRNA rescue is the most reliable approach to examine the specificity of the effects of MO knockdown. To rescue the gene-specific phenotype, co-injection of the MO of interest and synthetic mRNA encoding the targeted protein can be done at the 1–4 cell stage of the embryo (Hyatt and Ekker, 1999; Bedell et al., 2011). many genes, knockdown by MOs followed by ubiquitous mRNA delivery rarely results in truly rescued phenotypes (Bedell et al., 2011).The most important issues to consider in RNA rescue experiments are: (1) achievement of appropriate levels of injected MO and mRNA by co-injecting different concentrations of both components (Little and Mullins, 2004), (2) making sure that injected synthetic mRNA does not include the MO target sequence (Eisen and Smith, 2008). For TB MOs against the 5′ UTR sequence, the open reading frame can be cloned by PCR into a standard transcription vector (Hyatt and Ekker, 1999). For MOs that target part of the open reading frame, the rescue constructs can be engineered to change the nucleotide sequence without altering the encoded protein through degradation of the genetic code (Bill et al., 2009). Another frequently used approach for rescuing TB morphant is co-injection of mRNA from a different species. In their ZF model for PXE, Li et al. (2010) injected full-length mouse mRNA together with the MO; this co-injection completely reversed the phenotypic effects of the MO and the rescued embryos showed essentially the same morphology as controls. Co-injection of SJ MO and full-length mRNA (whose sequence is non-homologous to the SJ MO) can rescue a SJ morphant (Cline et al., 2012).

#### Qualitative and quantitative validation methods

The effect of TB MOs can be validated by western blotting, which correlates reduced protein levels of the targeted gene with an observed phenotype (Hutchinson and Eisen, 2006). The main obstacle for performing western blot in ZF is the currently limited availability of antibodies that are specifically generated to recognize ZF proteins, although a number of already available antibodies from other origins show cross-reactivity with ZF proteins.

**Figure 3:**
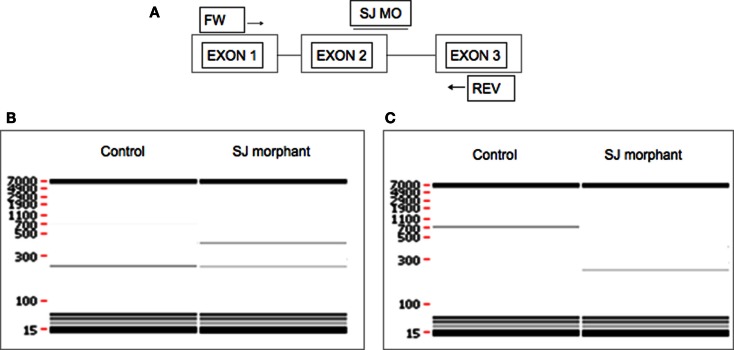
**Evaluation of the effect of SJ MO in the PCR product**. Hypothetical representation of intron retention and exon skipping as a consequence of SJ MO injection. **(A)** MO is placed on exon2/intron2 border on targated genes pre-mRNA. The result can show **(B)** retention of intron 2, or **(C)** skipping of exon 2. FW, forward primer; REV, reverse primer.

When using SJ MO, the phenotype can be validated with PCR. Qualitative evaluation of exon skipping or intron retention can be done by performing RT-PCR with primers located in exons upstream and downstream of the MO-targeted sequence showing smaller and larger band respectively in addition to the original band (Figure 3). Intron retention or exon skipping can result in a frameshift and consequently nonsense-mediated decay and reduced transcript levels can be assessed quantitatively by using quantitative real-time PCR (qRT-PCR).

## Characterization of (Ectopic) Mineralization Phenotypes

### Calcein staining

**Figure 4:**
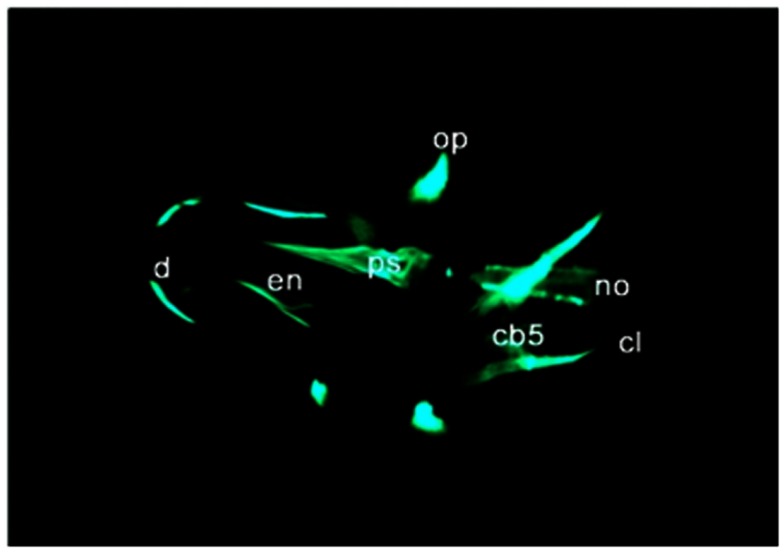
**Calcein staining of 6 dpf embryos showing staining of the ceratobranchials 5 (cb5), cleithrum (cl), dentary (d), entopterygoid (en), opercular bone (op), and parasphenoid (ps), in the skull, and anterior tip of the notochord (no)**.

Calcein (C_30_H_26_N_2_O_13_) is a fluorescent chromophore that specifically binds to calcium. As the skeletal system contains calcified structures, calcein has been used to label bone structures and to study bone growth (Ducy et al., 2000). During calcein staining, fluorescent chromophores of calcein rapidly penetrate into ZF embryos and specifically bind to the calcified skeleton (Figure 4). Calcein staining can be used to follow the development of skeletal structures in ZF embryos. Calcified skeletal structures appear in a progressive fashion from head to tail. First appearance of calcein signals, observed at ∼5 dpf are restricted to the head, followed by the axial skeleton in the trunk (Du et al., 2001). Du et al. (2001) observed that the axial skeleton calcified in two domains. This was later confirmed by AR-S staining (Bensimon-Brito et al., 2012). The first domain consists of three anterior vertebral centra (centra 3–5), whereas the second domain consists of the remaining abdominal and caudal centra which develop in an anterior-to-posterior direction. This confirms the sensitivity of calcein staining for visualizing mineralized structures in developing ZF embryos and its effectiveness for detecting defective bone structures and mineralization.

### Alizarin Red S staining

**Figure 5:**
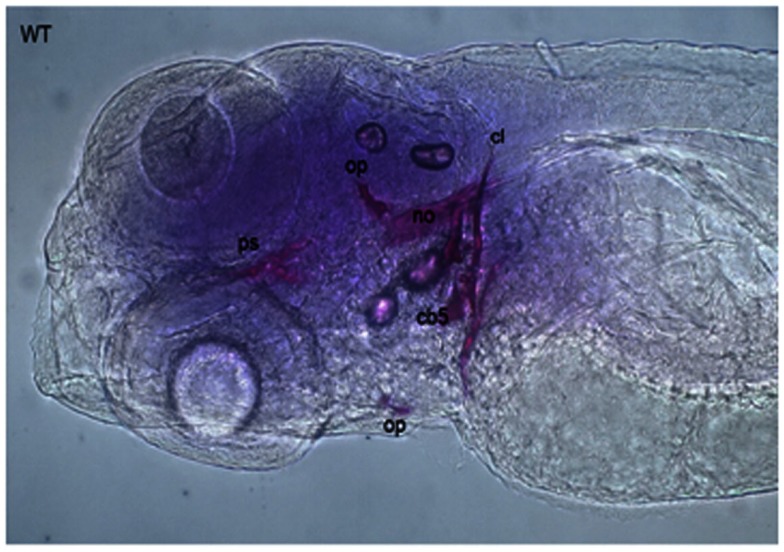
**Alizarin Red staining of a 5 dpf embryo showing cleithrum (cl), opercular bone (op), parasphenoid (ps), and ceratobranchials 5 (cb) with a set of three teeth**.

Alizarin Red S is an anthraquinone derivative used to identify calcium in tissue sections or whole mount embryos, where tissue calcium forms an AR-S-calcium complex in a chelation process, producing a birefringent end product (Nejati-Yazdinejad, 2006). The reaction is not strictly specific for calcium, since magnesium, manganese, barium, strontium, and iron may interfere, but these elements usually do not occur in sufficient concentration to interfere with the staining (Lievremont et al., 1982). AR-S staining can be performed on both live embryos and adult ZF as well as on fixed tissues. In live embryos, AR-S is used as a vital stain which labels mineralized matrix and is a very sensitive method for detecting bones (Kimmel et al., 1995; Walker and Kimmel, 2007). On the other hand, in fixed embryos or adult fish, the fixation itself is a very critical step for good AR-S staining. As PFA negatively affects bone staining, fixation with 4% PFA should be restricted to 2 h (Figure 5; Huitema et al., 2012) demonstrated an ectopic mineralization phenotype in dragon fish (dgf) mutants by Alizarin red staining, which exhibit ectopic mineralization in the craniofacial and axial skeleton and encode a loss-of function allele of ectonucleotide pyrophosphatase phosphodiesterase 1 (enpp1).

Besides AR-S staining, an Alcian Blue-AR-S double staining can be used to distinguish cartilage and bone. Walker and Kimmel (2007) developed an acid-free alcian blue-AR-S staining method which is now widely used to stain cartilage and bone simultaneously in ZF larvae.

### μCT imaging

**Figure 6:**
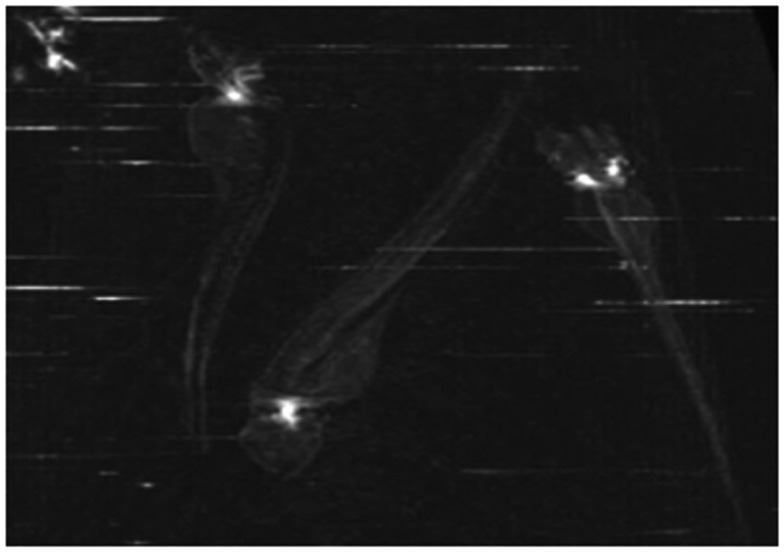
**MicroCT scanning of whole 4 dpf embryos**. The white spot in the head region represents a signal for the mineralized otoliths.

Micro computerized tomography (μCT) is a powerful and practical tool for the skeletal analysis of diverse model organisms including ZF. It is used to understand developmental processes of three-dimensional embryos, embryo phenotyping, and quantitative modeling of development (Figure 6) (Metscher, 2009). The method is analogous to that used for the 3D imaging of human structures, on a smaller scale. It is dependent upon the interaction of large atoms with X-ray beams and requires the use of contrast agents, still in development, for imaging anything other than bone. But the usefulness of μCT imaging for developmental biology has been limited by the low inherent contrast of embryonic tissues. Though it can be envisaged that μCT may also be useful to demonstrate ectopic calcification in, e.g., soft tissues, its limited sensitivity in embryos makes it a more useful technique in adult fish and hence the characterization of mutants instead of morphants. The ZF is the only well-developed vertebrate genetic model that is small enough to image the whole animal at cell resolutions using μCT (Cheng et al., 2011).

## Molecular Characterization

### Immunohistochemistry

Immunohistochemistry (IHC) is a powerful and commonly used technique in ZF for determining both the presence and localization of antigens (e.g., endogenous protein) in cells of a tissue section, or whole mount embryos and larvae. Sample preparation, especially sectioning and fixation of tissue, is very critical to maintain cell morphology, tissue architecture and the antigenicity of target epitopes. Non-specific binding can cause high background staining which may mask the detection of the target antigen. It is also possible to label both whole mount specimens and sections with two different antibodies, known as the double-labeling technique. Double-labeling works particularly well with fluorescent conjugated secondary antibodies and these can be applied at the same time.

Immunohistochemical approaches to reveal the localization of some calcification-related proteins demonstrated tissue distribution and accumulation of Bgp and Matrix γ-carboxyglutamic acid (Gla) protein (Mgp) during the larval stage and in adult tissues of ZF by IHC shows that Bgp and Mgp proteins were located in all mineralized tissues during and after calcification including bone and calcified cartilage of branchial arches (Gavaia et al., 2006). Being a powerful method to identify specific proteins by using an antibody, the main obstacle is the availability of antibodies and the limited cross-reactivity of ZF protein with antibodies originating from other sources.

### Whole mount RNA *in situ* hybridization

Whole mount RNA *in situ* hybridization (ISH) is one of the oldest and most frequently used techniques used in the ZF research to investigate the site of expression of a particular gene in an intact embryo (Jowett and Lettice, 1994). ISH allows specific nucleic acid sequences to be detected in morphologically preserved cells or embryos and is also used to identify novel genes involved in the same signaling pathways (Thisse and Thisse, 2008). Whole mount mRNA ISH can also be done to observe MO mediated gene expression changes. In this case, cDNA of a gene of interest is used as a template for the synthesis of an anti-sense mRNA probe, which is used to recognize and bind to the endogenous transcript through a color or fluorescence-based assay (Thisse and Thisse, 2008). Finally, the expression of the gene can be observed under a light or fluorescent microscope.

The major advantage for using the ISH technique in ZF compared to other animal models is that, because of its transparency and small size, the expression of a particular gene can be observed in the entire embryo. Though whole mount ISH is a quick and efficient method, the most significant caveat in ZF is the poor penetration of the RNA probes after 2 days of development; only superficial tissues (i.e., epithelial cells) are accessible to the probe at these later developmental stages (Thisse and Thisse, 2008). To overcome this, embryos can be treated with proteinase K with optimal concentration, incubation time and temperature for the embryonic stage to facilitate infiltration of the probes into the tissue.

ISH was performed to study the expression of the abcc6 gene (respectively the abcc6a and abcc6b isoform) during ZF development. While abcc6a was found to be expressed in Kupffer’s vesicle, abcc6b expression was evident in the proximal tubules of the embryonic kidney (Li et al., 2010).

In general, ISH is a laborious technique, taking about 3 days to complete the whole protocol. Additionally, it is difficult to handle small embryos because of their fragility throughout the protocol. So, semi-high-throughput procedures can alternatively be used to facilitate these experiments by using multiwell plates (instead of using single tube) and robotics (e.g., IntavisS AG) (Thisse and Thisse, 2008; Bouzaffour et al., 2009). To detect the differential protein expression in morphant compared to control, ISH followed by image mapping software can be a useful tool. Alternatively, post-ISH embedding in plastic, followed by sectioning, yields high resolution images that allow detailed cellular localization of the transcripts (Verstraeten et al., 2012).

### Assessment of gene expression using quantitative real-time PCR and microarrays

Real-time polymerase chain reaction, also called qPCR, is a laboratory technique based on PCR, which is used to detect and measure minute amounts of nucleic acids in a wide range of samples. The quantity can be either an absolute number of copies or a relative amount. The expression pattern of mineralization-related genes in morphants (i.e., osteocalcin, alkaline phosphatase, bone sialoprotein) can be assessed by performing quantitative RT-PCR. To reliably conduct qRT-PCR experiments it is highly recommended to follow the MIQE guidelines (Bustin et al., 2009). MIQE is a set of guidelines that describe the minimum information necessary for evaluating qPCR experiments. Among the requirements is the need to include at least three biological replicates (e.g., three independently injected clutches of embryos) to address the statistical significance of differences in qPCR results between morphants and controls. Furthermore, at least two reference genes should be included as internal controls for normalizing cellular mRNA data. Reference genes should be stably expressed, and their abundances should show strong correlation with the total amount of mRNA present in the samples. In ZF embryos tuba1, bactin1, and elfα are three stable reference genes which can be used for qPCR experiments comparing morphants and controls (McCurley and Callard, 2008).

Microarray technology can be used to monitor transcriptome wide expression of genes in ZF embryos. Microarray analysis has been employed to study the temporal activity of developmentally regulated genes during ZF embryogenesis (Mathavan et al., 2005) but also in numerous studies where gene expression was compared between morphants and controls (Jenny et al., 2012; Wei et al., 2012).

The principle of Next Generation Sequencing (NGS) has recently been applied to transcriptome profiling (Wang et al., 2009), which offers several advantages compared to microarrays or quantitative RT-PCR. Most importantly, RNA-Seq transcriptome profiling can be used to identify rare transcripts which are undetectable with microarrays. Moreover, RNA-Seq allows more precise quantification of different transcripts (Wetterbom et al., 2010).

### Proteomics

Physiological mineralization is governed by highly coordinated changes in the expression of a large number of proteins. Understanding these changes at the molecular level can provide important insights into physiological and disease mechanisms (Lucitt et al., 2008). To elucidate these regulatory genetic networks in a ZF disease model, quantitation of protein expression during growth and development is essential. In morphants, proteomics can be used to identify proteins that are differentially expressed compared to controls. It has been proposed that proteomic studies should complement genome-wide expression profiling (Love et al., 2004).

Many approaches can be used for quantitative protein studies including two dimensional poly acrylamide gel electrophoresis (2D PAGE), mass spectrometry (MS), liquid chromatography (LC) and western blotting. Proteomic approaches are however incrementally challenging in ZF because of unavailability of specific antibodies and the high abundance of yolk proteins in embryos (Akhtar et al., 2009; Lobner et al., 2012). Further, many protein identifications have low reproducibility if the sensitivity of detection is not carefully balanced against rates of false identification error (Lucitt et al., 2008). Therefore, rigorous statistical analysis is needed to obtain high quality profiles of proteins.

In the early embryo, the cells forming the embryo constitute only a minor volume compared to the large yolk sac. The major yolk protein is vitellogenin, a phospholipo-glycoprotein, which functions as a nutritional source for the development of the embryo (Denslow et al., 1999). Link et al. (2006) developed an effective protocol for protein analysis from deyolked embryos. By pipetting with a narrow tip, the yolk sac can be disrupted. A buffer of low osmolarity facilitates dissolving of the yolk. The deyolking efficiency can be further increased by two additional wash steps. By removing the yolk, recovery of cellular proteins remained high with only a minor reduction of housekeeping gene (mek and tubulin) observed.

#### Mass spectrometry

Mass spectrometry is an important method used to characterize proteins by measuring the mass-to-charge ratio. The first step in MS is the ionization of proteins, for which two common methods can be used: electrospray ionization (ESI) and matrix-assisted laser desorption/ionization (MALDI). Mass analysis of proteins can be conducted using either time-of-flight (TOF) MS or Fourier transform ion cyclotron resonance (FT-ICR). Generally, a protein sample is a complex mixture of different proteins. The concentration of a protein can vary between samples and an overwhelming number of peptides can make it difficult to interpret the results. To overcome this problem, two methods, 2D gel electrophoresis and high performance liquid chromatography, can be used to fractionate the proteins or their peptide products after enzymatic digestion. In MS, two ways are mainly used to identify proteins, i.e., Peptide mass fingerprinting and Tandem MS. Observed fragment masses are matched with a database of predicted masses for given peptide sequences. Several methods also allow quantitation of proteins by MS, i.e., stable isotope labeling by amino acids in cell culture (SILAC), isotope coded affinity tagging (ICAT), iRRAC (isobaric tags for relative and absolute quantitation), or semi-quantitative MALDI analysis (in liner mode).

#### 2D gel electrophoresis

After removal of the predominant yolk proteins, high resolution 2D gels in the acidic (pH 4–7) as well as in the basic range (pH 6–9) can be run, and proteins will be separated according to isoelectric point. Two biological replicates of each sample have to labeled with fluorophores (Cy3/Cy5), and an internal control from each sample labeled with different fluorophores (Cy2) can be used to normalize label differences. DIGE gels are then stained and imaged using emission wavelengths of the fluorophores. Link et al. (2006) established a protocol that is compatible with three color fluorescent labeling using the Ettan DIGE system, which significantly reduces inter-gel variability compared to one color stains with a detection limit less than 1 ng protein. Analysis of 2D gels also allowed to resolve protein isoforms. Finally, validation of protein expression changes can be confirmed by western blotting and real-time PCR studies.

#### Western blotting

Western blot is a widely accepted analytical technique used to detect specific proteins in a given sample of tissue homogenate or extract. The success of western blotting depends on the affinity and specificity of the antibodies and on the abundance of the target protein. If the yolk sac is not removed only 1 or 2 embryos (50–100 μg) can be loaded per lane on a gel to avoid overloading effects (Link et al., 2006). As previously stated, antibodies validated for ZF proteins are not always available which can hamper the use of this technique. Along with confirmation of TB MO efficiency, western blotting can also be used to detect differential expression of a targeted protein in morphant compared to control.

## Disease Specific Mechanisms

### Identification of programed cell death (apoptosis)

Apoptosis is a form of cell death in which a programed sequence of events leads to the elimination of cells without inflammation. Apoptosis plays a pathophysiological role in many mineralization-related disorders including calcific aortic valve disease (Côté et al., 2012), osteoarthritis (Sun et al., 2012), and PXE (Mungrue et al., 2011).

**Figure 7:**
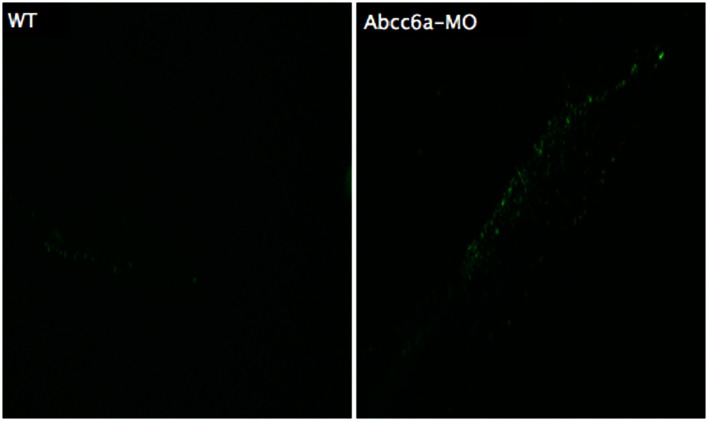
**Transferase dUTP nick end labeling staining of 5 dpf embryos**. More fluorescent dots are observed in the tail region of Abcc6a-MO injected fish (right) demonstrating more apoptosis compared to the tail region of the wild-type fish (left).

Several methods have been developed for visualizing apoptotic cells *in vitro* or in fixed tissues, but few tools are available for visualizing apoptotic cells in live animals. Methods exist for labeling apoptotic cells with fluorescent nucleic acid binding dyes, such as acridine orange, ethidium bromide, and propidium iodide (Lecoeur et al., 2002). A standardized technique to detect apoptotic cells in fixed tissue or fixed cells is terminal deoxynucleotidyl TUNEL, which is based on end labeling of DNA degradation products enzymatically or by a fluorescent probe (Figure 7) (Gavrieli et al., 1992). Another well-established method to detect apoptotic cells *in vitro* is based on loss-of membrane asymmetry during apoptosis (Fadok et al., 1992). During apoptosis, the normal asymmetric distribution of phospholipids in the cell membrane is lost, and phosphatidylserine (PS) is exposed on the outer leaflet of the lipid bilayer. The calcium-dependent protein Annexin V (A5) binds PS with high affinity and fluorescently labeled A5 probes have been widely used to detect apoptotic cells *in vitro* (Van Genderen et al., 2006).

Van Ham et al. (2010) recently introduced a new transgenic fluorescent marker allowing *in vivo* imaging of apoptotic cells to understand their dynamics. They fused secreted A5 (secA5) protein to yellow fluorescent protein (YFP) (secA5-YFP) and showed that this fusion product specifically labels apoptotic cells in living ZF; the fluorescent probe can characterize patterns of apoptosis in living ZF larvae and visualize cell death at single-cell resolution *in vivo*. Labeled cells exhibit several other characteristics of apoptotic cells, and the pattern of apoptotic cells observed by live imaging was similar to previous findings using TUNEL.

### Detection of oxidative stress

Oxidative stress is an imbalance between the systemic manifestations of reactive oxygen species (ROS) and a biological system’s ability to readily detoxify the reactive intermediates or to repair the resulting damage. Disturbances in the normal redox state of cells can cause toxic effects through the production of peroxides and free radicals that damage all components of the cell, including proteins, lipids, and DNA. Oxidative stress is increasingly implicated as a possible pathogenic mechanism underlying a wide range of diseases including mineralization-related disorders such as atherosclerosis, cardiovascular disease, or diabetes (Stephens et al., 2009). Oxidative stress in fibroblasts (Pasquali-Ronchetti et al., 2006), and elevated oxidative stress markers in the circulation (Garcia-Fernandez et al., 2008) of patients with PXE reveal its possible role in the pathogenesis of this ectopic calcification disease.

To determine the level of ROS, fluorescent reporter molecules CMH2DCF and Dihydrorhodamine can be administered, both of which have been successfully applied to live embryos (Hermann et al., 2004; Craven et al., 2005). Dihydrorhodamine is an uncharged fluorescent ROS indicator which passively diffuses across membranes where it is oxidized to cationic rhodamine 123 which localizes in the mitochondria and exhibits green fluorescence (Song et al., 2009).

### Analysis of mitochondrial membrane potentiality

Recent findings show that besides the production of ATP, mitochondria also contribute to several other cellular functions, including redox homeostasis, calcium homeostasis, and cell death (Scheffler, 2001). Mitochondrial mutations (inherited or somatic) are responsible for many developmental abnormalities (Zhang et al., 2012). Mitochondrial dysfunction also plays an important role in many mineralization diseases; i.e., in PXE and vascular calcification where decreased mitochondrial membrane potentiality (MMP) and reduced ion gradient has been reported. Measuring MMP allows assessment of mitochondrial function and integrity (Nicholls and Budd, 2000).

**Figure 8:**
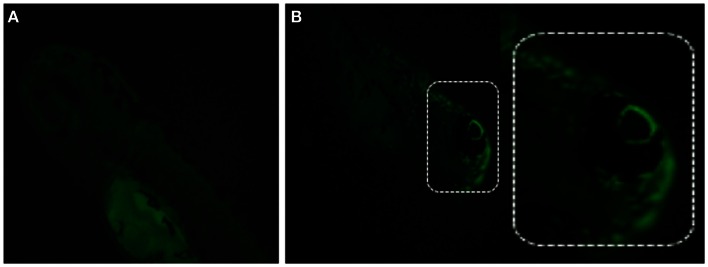
**Detection of mitochondrial membrane potentiality on 2 dpf embryos by MitoTracker Red CM-H2XRos staining**. **(A)** Control fish showing no fluorescent staining, and **(B)** staining with 500 nM MitoTracker Red CM-H2XRos for 2 h showing fluorescent staining of mitochondria in the head region of a mitochondrial disease model.

Different methods exist to measure MMP in ZF, including staining with membrane-potential-dependent dyes such as Rhodamine 123, tetramethylrhodamine ethyl ester (TMRE), fluorescent carbocyanine dye JC-1, or MitoTracker (Chazotte, 2010; Mitra and Lippincott-Schwartz, 2010). MitoTracker is a commercially available fluorescent dye, which labels mitochondria within live cells. Among available probes, MitoTracker Red is a red-fluorescent dye widely used for labeling mitochondria in live ZF embryos/cells and its accumulation depends upon membrane potential (Figure 8). Live ZF embryos are incubated in the dark with 25–500 nM MitoTracker Red CM-H2XRos working solution for 2 h at 28.5°C, and observed under a fluorescent microscope. MitoTracker can also stain the endoplasmic reticulum if embryos/cells are exposed to a higher MitoTracker solution for a prolonged period of time. However, high concentration and prolonged exposure can also block mitochondrial activity, so a low concentration with short time exposure is recommended to obtain specific staining (Ryu et al., 2011).

### Chemical screening and drug discovery

Due to easy diffusion of chemicals through the skin, ZF allows disease-driven drug target identification and *in vivo* validation, thus representing an interesting bioassay tool for small molecule testing and dissecting biological pathways (Pichler et al., 2003). In a MO injection based reverse genetic approach, chemical screening is very useful to find a chemical/drug which rescues morphant phenotypes (Taylor et al., 2010). Peterson et al. (2000) screened 1100 synthetic small molecules against ZF embryos arrayed in 96 well plates to identify molecules that specifically modulated developmental processes, and found that one tetrazole derivative affected otolith development. By adding and removing the chemical, they determined a critical stage for otolith development that occurs between 14 and 26 h after fertilization. This demonstrates that drug screening in ZF can provide more insights in physiological and developmental processes.

Pichler et al. (2003) proposed two broad strategies for drug development in ZF. First, large-scale random chemical screening against diseased or control ZF can be performed to observe biologically interesting phenotypic changes; secondly, functional understanding of disease pathways can allow to determine specific target genes directly associated with the disease followed by chemical screening against that gene. In combination with microarray, ZF promise to be a cost and efficient bioassay that can simultaneously uncover drug candidates, estimate toxicity, primary and secondary drug targets, and phenotypic outcomes (Pichler et al., 2004). As many ectopic mineralization disorders are currently still intractable, this model may prove efficient to further explore therapeutic options.

## Systems Biology Approaches

The multidisciplinary approaches of systems biology allow us to quantitatively study of the fundamental principles of a biological system, aiming at better understanding the connections between gene, protein, and cellular networks. ZF is regarded as an ideal vertebrate model for studying genotype-phenotype relationships, pathway analysis, and systems biology. Deo and MacRae (2011) described the possibility of a systems biology approach in ZF for functional annotation of the genome, elucidation of multicellular processes *in vivo*, expression profiling and genome-wide association, phenotypic architecture, disease heterogeneity, and drug responses. Several systems biology approaches have already been developed to study ZF models; among them, the comparative study of embryonic development between morphants and controls can break new ground in understanding and uncovering disease mechanisms. However, the bottleneck of these approaches is the huge amount of data that needs to be analyzed.

### In toto imaging

Megason and Fraser (2007) recently described “Digital Fish project” to upload ZF development into computer for better understanding how genome computes the formation of an embryo from an egg. In their project they combine confocal/2-photon imaging with genetics, genomics, synthetic biology, and computational analysis to watch biological circuits function *in vivo* and use these data in cell-based modeling. They developed “in toto imaging” to track all cells in a developing tissue and record cell-based quantitative data by using fluorescent reporters. In this imaging process they used “segmentation marker” to track cells, captured 3D time-lapse movies on confocal and custom built 2-photon microscope, and finally used a software package called GoFigure to determine complete lineages and cell-based frameworks for use in modeling.

### Flip traps

Flip traps is a novel gene trap technique to generate endogenously expressed functional fluorescent fusion protein, and they generate Cre conditional alleles. Fluorescent fusion protein is used to non-invasively quantify expression and localization of proteins *in vivo*. Flip Traps in combination with “in toto imaging” can digitize expression and phenotype with single-cell resolution for use in molecular and cellular modeling of developmental processes (Megason and Fraser, 2007).

### Computational approach

Morelli et al. (2012) recently described the use of computational modeling (algorithm and simulation) to understand embryonic development. They investigated four developmental patterning strategies of the embryo: (1) gradients of signaling molecules released from localized source cell population, (2) balance between activator-inhibitor mechanism leading to formation of spatial patterns, such as stripes and spots in a two dimensional space, (3) synchronization of cellular oscillations controlling rhythmic and sequential subdivision of body axis into segments, and (4) mechanical deformation changing the pattern of a cellular population. It is shown that theoretical along with experimental/computational data can play an important role to disclose mechanisms of development. Following the same approach between control and morphant embryos will give an opportunity to understand underlying differential mechanisms. The Prerequisite for using this approach is that the level of description and model type are matched to quantitative, precise, and accurate data. This will beyond any doubt require a multidisciplinary team of specialists.

### *In vivo* modeling

Genetic and genomic features, conservation of intermolecular network, as well as physiologic and phenotypic features have made ZF an important model for systems biology studies. Deo and MacRae (2011) described homology and high-throughput phenotyping strategies which can be used for genetic or chemical screening on a scale compatible with *in vivo* validation for systems biology.

Advent and validation of MO and increasing efficiency of transgenesis have made it possible to study hundreds of genes with specific phenotypes. Generally, phenotypic investigation of these approaches has been limited by phenotyping throughput. Now, the possibility of automated, quantitative phenotypes can lead to more comprehensive screenings. Moreover, applying a known causal mutation background in ZF models can aid to uncover disease mechanism. This type of analysis is also very useful to understand genotype-phenotype correlations including penetrance, pleiotropy, and pathogenicity. ZF disease models combined with known/approved libraries of drugs may enable collection of datasets which can be highly informative not only for disease network architecture, but also for pharmacogenetics.

## Conclusion

**Figure 9:**
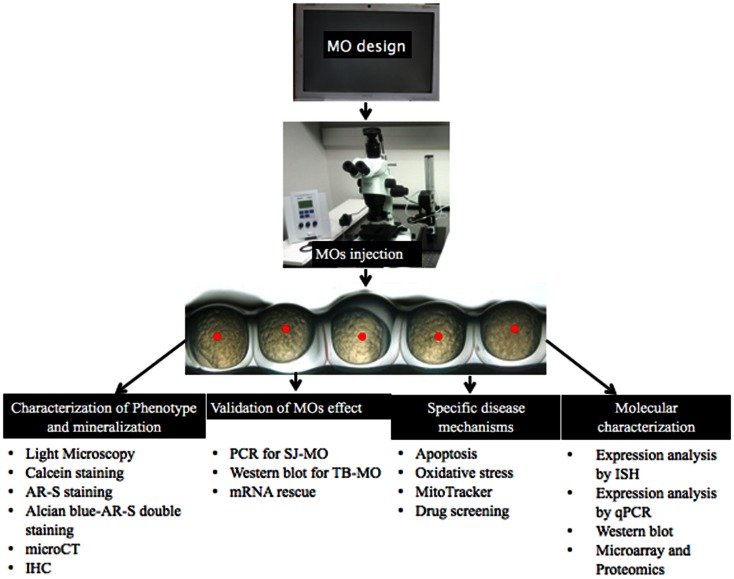
**Overview of MO injection and post-injection follow-up to study ectopic mineralization zebrafish models**.

Ectopic mineralization disorders feature some important medical issues to be resolved because of their complex pathogenesis, uncertainties on the mechanisms that deposit mineral in tissues or how to remove it from the tissues, and the unavailability of specific drugs and treatments. Until now, many experiments have been done both on patients and mammalian model organisms but the pathophysiological mechanisms of many ectopic mineralization disorders are still incompletely understood. Because of the limitations to perform studies on human tissues – often due to the invasive procedures needed to obtain them – and the high cost, long breeding time and complexity to achieve knockdown of genes in mouse models, the ZF has come to attention as an alternative model organism. Several studies have shown that there are many similarities in the molecular pathways and mechanisms involved in (pathological) mineralization between ZF and mammalians, even if the phenotypic consequences are not identical for obvious reasons. Using MO injection based knockdown, ZF can be an important disease model to study mineralization disorders. This is because phenotypic and molecular genetic results can be obtained within hours, because of the possibility of close observation in the developing transparent embryos and because of the easy application of techniques in the post-injection period. The concern of off-target or aspecific findings can be addressed by applying one or more alternative knock-down approaches. By considering aforementioned advantages, ZF can be used as a novel model organism for ectopic mineralization disorders. After MO-based knockdown, the described (Figure 9) validation and post-injection follow-up can be applied to gain insights in the mechanisms and future therapeutics of mineralization disorders.

## Conflict of Interest Statement

The authors declare that the research was conducted in the absence of any commercial or financial relationships that could be construed as a potential conflict of interest.

## Footnotes

^1^http://research.nhgri.nih.gov/ZInC/

^2^http://www.sanger.ac.uk/Projects/D_rerio/zfm_DAS_conf.shtml
